# 789 Role of Speech Therapy (SLP) in the rehabilitation of a complicated case of glossoparesis (GP)

**DOI:** 10.1093/jbcr/irac012.340

**Published:** 2022-03-23

**Authors:** Janet Shaw, Joan Wilson

**Affiliations:** Joseph M. Still Burn Center at Doctors Hospital, Augusta, Georgia; Joseph M. Still Burn Center at Doctors Hospital, Augusta, Georgia

## Abstract

**Introduction:**

Glossoparesis is defined as a neurodegenerative process resulting in progressive dysphagia and dysarthria. The deinervation of the tongue and related structures results in flaccid muscle function unilaterally and bilaterally, this process is difficult to treat and rehabilitate and prognosis is considered poor. We encountered this in a patient that had a complicated case of necrotizing fasciitis (NF) that arose from an abscessed tooth.

The purpose of this case report is to summarize the overall approach to a rehabilitation plan which resulted in successful return to speech and partial swallow.

**Methods:**

The patient was diagnosed with an abscessed tooth which resulted in Necrotizing fasciitis of the face, neck and chest. Multiple surgical interventions resulted in removal of necrotic tissue and reconstructive interventions were implemented. Initial prognosis of return of function was noted as “poor” per ENT services. Ear, Nose and Throat (ENT) specialist noted that prognosis for recovery of function is unknown but was low.

Speech Therapy was also consulted and an individualized plan of care to address the deficits assessed was developed. Initial prognosis of return of functional speech / swallow was guarded. It was felt to be highly appropriate to provide the patient with therapeutic options.

Speech Therapy sessions included use of Neuromuscular Electrical Stimulation, Thermal Stimulation, Tactile Stimulation, Therapeutic Massage to affected regions, Deep Pharyngeal Neuromuscular Stimulation, finger occlusion trials for speech production attempts to ready patient for use of Passy Muir Valve, Oral Motor Exercises, Laryngeal Elevation Tasks, Vocal Stretching / Relaxation tasks and oral trials of ice chips as musculature function improved.

**Results:**

Through the use of these multiple modalities of skilled speech therapy intervention, the patient had return of functional verbal communication prior to discharge to a skilled rehabilitation program.

**Conclusions:**

Upon initial evaluation, the true potential for return of function was questioned. It is paramount that patient be given an opportunity to succeed - to be included in the plan of care - to be provided with opportunities to return to their prior level of function or as close as possible. There is little to no research regarding return of function for patients affected by glossoparesis.

